# Assessment of perioperative anesthesia record sheet completeness: A multi-center observational study

**DOI:** 10.1016/j.amsu.2022.104103

**Published:** 2022-07-02

**Authors:** Moges Gelaw Taye, Efrem Fenta, Tadese Tamire, Yewlsew Fentie

**Affiliations:** Department of Anesthesia, College of Health Sciences, Debre Tabor University, Debre Tabor, Ethiopia

**Keywords:** Anesthesia record completeness, Perioperatively, Ethiopia, DCSH, Dessie Comprehensive Specialized Hospital, DTCSH, Debre Tabor Comprehensive Specialized Hospital, ECG, Electro Cardiography, FHCSH, Felege Hiwot Comprehensive Specialized Hospital, WHO, World Health Organization

## Abstract

**Background:**

Anesthesia record is an essential part of perioperative patient management and, it is one of the global patient safety challenges. The aim of this study is to assess the completeness of perioperative anesthesia record sheets in Amhara regional state hospitals of Ethiopia.

**Methods:**

A retrospective review of anesthetic records was employed for patients who underwent surgery in Amhara Regional State Hospitals of Ethiopia from December 1, 2019, to February 29, 2020. All the intraoperative, preoperative, and postoperative data completeness of anesthesia record sheets was assessed using a structured checklist.

**Results:**

A total of 420 perioperative anesthetic records were included in this study. The basic preoperative history was documented in less than 80% of anesthesia record sheets. A physical examination was done and the results of the basic investigations were recorded in less than 75% of anesthesia record sheets. The name and dose of anesthetic medications were documented in 91% of the anesthesia records sheets. The completeness of parameters related to postoperative plans and orders was less than 70%.

**Conclusion:**

The completeness of perioperative anesthesia record was poor in Amhara regional state hospitals of Ethiopia. Anesthesia professionals should document all the necessary perioperative parameters in the anesthesia record sheet.

## Highlights

• Anesthesia record is global patient safety challenge.
• Anesthesia record was poor in Amhara regional state.
• Anesthesia professionals should be alarmed on anesthesia record.

## 1 Introduction

The anesthetic record is an essential part of perioperative patient management. It is one of the Global Patient Safety Challenge. The expertise of anesthesia recording completeness helps to improve the safety of surgical care [[1], [2], [3]]. The anesthetic record should be recorded in a simple and logical manner, either electronically or by handwritten document [4]. The anesthetic record helps to document all the important perioperative data. Documented records can serve as legal documents during the legal liability of anesthesia professionals. It is also used to maintain a safe practice of anesthesia and for research purposes [5].

The World Health Organization-World Federation of Societies of Anesthesiologists (WHO-WFSA) states the basic components of the perioperative anesthesia record sheets to maintain international standards for a safe practice of anesthesia [6]. The basic components of the anesthetic record sheet include patient-related data, preoperative assessment, provider information, anesthetics drugs and monitors, anesthetic techniques, perioperative vital signs, postoperative orders, and patient outcomes [[5], [6], [7], [8], [9], [10]].

Despite the presence of guidelines, the completeness of perioperative documentation has not been adequately assessed. The lack of adequate perioperative documentation has legal implications and can potentially affect the quality and safety of patient care [6,11]. As far as our knowledge there were no studies conducted that show the completeness of anesthetic record sheets in the selected study settings. The purpose of the current study was to assess the completeness of perioperative anesthesia record sheets.

## 2 Methods

### 2.1 Study settings and period

The study was conducted in Debre Tabor, Felege Hiwot, and Dessie Comprehensive Specialized Hospitals. These hospitals were selected using lottery method from eight Comprehensive Specialized Hospitals in Amhara regional state of Ethiopia. All of these Hospitals have operation rooms that give services on elective and emergency bases. Approximately 15,000–20,000 patients undergo surgery in Amhara regional state Hospitals per year. This retrospective review of anesthetic records was conducted from December 01, 2019, to February 29, 2020. This study is registered at http://www.researchregistry.com with Research Registry UIN: researchregistry7687. The manuscript is also reported according to STROCSS criteria [12].

### 2.2 Source population

All anesthetic records of patients who underwent surgery in Amhara Regional State Governmental Hospitals of Ethiopia.

### 2.3 Study population

Selected anesthetic records of patients who underwent surgery in selected Amhara Regional State Governmental Hospitals of Ethiopia during the study period.

### 2.4 Sample size and sampling technique

The sample size was determined using the single population formula by taking the following assumption; the proportion of complete anesthesia record sheets of 53.9% from a previous study done in Nigeria [13], Confidence interval of 95% and margin of error of 0.05. The sample size was determined using the following single population proportion formula.

n=z2(p)(1−p)/d2

whereas; n = sample size; Z = confidence interval (1.96); P = prevalence (0.5); d = margin of sampling error to be tolerated (0.05). The sample size was calculated as follows with confidence interval of 95% and margin of error 5%.

n= (1.96)^2^ × 0.539(1–0.539)/(0.05)^2^ = 385, and by adding 10% non-response rate the final sample size was 420. This sample size was distributed proportionally to the three hospitals**.** The random sampling technique was used to select the anesthetic record sheets in each setting. Anesthetic record sheets were identified from Institutional Anesthesia record books and clinical records (case notes). The case notes and perioperative anesthesia record charts were retrieved from the records department of each Hospital.

### 2.5 Inclusion criteria

All perioperative anesthesia record sheets were included in the study based on the proportional allocation of samples for the selected Hospitals.

### 2.6 Exclusion criteria

Records that are damaged and difficult to identify the documented values were excluded.

### 2.7 Operational definitions

Poor anesthetic record: The American Association of Nurse Anesthetists states that any anesthesia records of any parameters with less than 95% are considered poor [9].

### 2.8 Data quality control

The checklist was pretested on 5% of samples at Debre Markos Comprehensive specialized Hospital before the actual data collection commenced. The training was given for data collectors; Close supervision was undertaken during the data collection, and the data were coded and put in a secured place.

### 2.9 Data collection technique and instrument

The anesthetic record sheets of selected hospitals were used to extract perioperative data. Then the trained anesthetists extract those perioperative data based on the prepared checklist. We used the nationally harmonized anesthesia record sheet as a standard checklist to evaluate the completeness of anesthetic records of the study sites against it. This record sheet was prepared by professional experts from different hospitals of Ethiopia. It is also in line with the American Association of Nurse Anesthetists. The checklist consists of data related to patient information, preoperative assessment, preoperative physical examination, preoperative investigations, intraoperative anesthetic-related data, intraoperative medications and monitoring used, and postoperative plans.

Data related to patient information: Name of the patient, age, sex, weight, medical registration number; Data related to the preoperative assessment: Date seen, planned procedure, preoperative diagnosis, the background to diagnosis, past medical history, history of surgical and anesthetic exposure, system inquiry, history of reflux, history of medication, allergic history, family history of medical illness, history of smoking/alcohol/other drugs, and last food/drink; Data related to preoperative physical examination: HEENT, oropharyngeal view, thyromental distance, jaw slide. neck movement, cardiovascular system, respiratory system, ASA physical status, informed consent was signed; Data related with preoperative investigations: Complete Blood count, Renal function tests, Blood group and Rh, Electrolytes, Chest X-ray, and ECG; Data related with intraoperative anesthetic record: Anesthetic machine-checked, the actual procedure is done, date of surgery, name of the qualified anesthetist, name of the surgeon, size, and site of iv cannula, intraoperative monitoring used, and type of anesthesia (regional/general); Data related with intraoperative medications and monitoring used: Oxygen flow rate, name, and a dose of premedication drugs, name, and a dose of anesthetic medications, intraoperative vital signs recorded clearly, total estimated blood loss, blood transfusion, urine output, total intravenous fluids, surgical duration, and anesthetic duration; and Data related with postoperative plans: Conditions in the recovery documented, postoperative nausea and vomiting plan, postoperative pain management plan, postoperative fluid management plan, and postoperative vital sign monitoring. The anesthetic records were selected by using simple random sampling technique.

### 2.10 Statistical analysis

The collected data were checked manually for completeness and entered into the SPSS version 23.0. Parameters related to the perioperative period and extracted from the anesthesia record sheets were described in terms of frequencies and percentages. The data were presented using tables.

### 2.11 Ethical considerations

Ethical clearance and approval were obtained from Debre Tabor University Ethical Review Committee. We obtained the permission letter to conduct the research from the Amhara Regional Health Bureau and the respective Hospitals. The data were coded to secure the confidentiality of patient information.

## 3 Results

### 3.1 Data related with patient information

A total of 420 anesthetic record sheets were assessed for completeness of perioperative data. Among those record sheets a total of 20 (4.76%) were filled by similar anesthetists. The highest filled number of record sheets by a single anesthetist was 16 (3.81%). The level of anesthetic record completeness was 95.5%, 90%, 77.4% and 90.2% for name, age, sex and medical registration number respectively. On the other hand, the completeness of documentation was <50% for weight (Table 1).

**Table 1:** Anesthetic record completeness about patient identity in Amhara Regional State hospitals of Ethiopia.

Parameters	Yes Frequency (%)	No Frequency (%)
Name of patient	401 (95.5)	19 (4.5)
Age	378 (90)	42 (10)
Sex	325 (77.4)	95 (22.6)
Weight	181 (43.1)	239 (56.9)
Medical registration number	379 (90.2)	41 (9.8)

### 3.2 Data related with the preoperative assessment of patients

Completeness of all the documented data related to basic preoperative history was less than 80% except for history of surgical &/anesthetic exposure and history of medication (Table 2). Most of the parameters related to preoperative physical examination are less complete (Table 3) and the record completeness of basic preoperative investigations was less than 75% (Table 4).

**Table 2:** Anesthetic record completeness about basic preoperative history in Amhara Regional State hospitals of Ethiopia.

Parameters	Yes Frequency (%)	No Frequency (%)
Date seen	280 (66.7)	140 (33.3)
Planned procedure	320 (76.2)	100 (23.8)
Preoperative diagnosis	322 (76.7)	98 (23.3)
Background to diagnosis	308 (73.3)	112 (26.7)
Past medical history	280 (66.7)	140 (33.3)
History of surgical and anesthetic exposure	336 (80)	84 (20)
System inquiry	244 (58.1)	176 (41.9)
History of reflux	256 (61)	164 (39)
History of medication	336 (80)	84 (20)
Allergic history	308 (73.3)	112 (26.7)
Family History of Medical Illness	320 (76.2)	100 (23.8)
History of smoking/alcohol/other drugs	279 (66.4)	141 (33.6)
Last food/drink	244 (58.1)	176 (41.9)

**Table 3:** Anesthetic record completeness about preoperative physical examination in Amhara Regional State hospitals of Ethiopia.

Parameters	Yes Frequency (%)	No Frequency (%)
HEENT	360 (85.7)	60 (14.3)
Oropharyngeal view	356 (84.6)	64 (15.2)
Thyromental Distance	323 (76.9)	96 (22.9)
Jaw slide	279 (66.4)	141 (33.6)
Neck movement	278 (66.2)	142 (33.8)
Cardiovascular System	306 (72.9)	114 (27.1)
Respiratory System	370 (88.1)	50 (11.9)
ASA physical Status	333 (79.3)	87 (20.7)
Informed consent was signed	345 (82.1)	75 (17.9)

HEENT: Head eye ear nose and throat.

**Table 4:** Anesthetic record completeness about basic preoperative investigations in Amhara Regional State hospitals of Ethiopia.

Parameters	Yes Frequency (%)	No Frequency (%)
Complete Blood count	244 (58.1)	176 (41.9)
Renal function tests	225 (53.6)	195 (46.4)
Blood group and Rh	278 (66.2)	142 (33.8)
Electrolytes	308 (73.3)	112 (26.7)
Chest X-ray	270 (64.3)	150 (35.7)
ECG	308 (73.3)	112 (26.7)

**Table 5:** Anesthetic record completeness about basic intraoperative anesthetic record in Amhara Regional State hospitals of Ethiopia.

Parameters	Yes Frequency (%)	No Frequency (%)
Anesthetic machine checked?	252 (60)	168 (40)
Actual Procedure done	310 (73.8)	110 (26.2)
Date of surgery	335 (79.8)	85 (20.2)
Name of qualified anesthetist	327 (77.9)	93 (22.1)
Name of surgeon	349 (83.1)	71 (16.9)
Size and site of IV cannula	338 (80.5)	82 (19.5)
Intraoperative monitoring used	336 (80)	84 (20)
Type of anesthesia (regional/general)	361 (86)	59 (14)

**Table 6:** Anesthetic record completeness about intraoperative medications and monitoring in Amhara Regional State hospitals of Ethiopia.

Parameters	Yes Frequency (%)	No Frequency (%)
Oxygen flow rate	360 (85.7)	60 (14.3)
Name and dose of premedication drugs	336 (80)	84 (20)
Name and dose of anesthetic medications	381 (90.7)	39 (9.3)
Intraoperative vital signs recorded clearly	321 (76.4)	99 (23.6)
Total estimated blood loss	267 (63.6)	153 (36.4)
Blood transfusion	292 (69.5)	128 (30.5)
Urine output	324 (77.1)	96 (22.9)
Total Intravenous fluids	319 (76)	101 (24)
Surgical duration	310 (73.8)	110 (26.2)
Anesthetic duration	328 (78.1)	92 (21.9)

### 3.3 Data related intraoperative medications and procedure

The name and dose of anesthetic medications were documented in 90.7% of the anesthesia records. Many of the intraoperative parameters are documented in less than 80% of the records (Table 5, Table 6).

### 3.4 Data related with postoperative plan

The completeness of parameters related to postoperative plans and orders is poorly documented (less than 70%) in most of the anesthesia records (Table 7).

**Table 7:** Anesthetic record completeness regarding postoperative plans in Amhara Regional State hospitals of Ethiopia.

Parameters	Yes Frequency (%)	No Frequency (%)
Conditions in the recovery documented	279 (66.4)	141 (33.6)
Postoperative nausea and vomiting plan	231 (55)	189 (45)
Postoperative pain management plan	234 (55.7)	186 (44.3)
Postoperative fluid management plan	249 (59.3)	171 (40.7)
Postoperative vital sign monitoring	239 (56.9)	181 (43.1)

## 4 Discussion

The anesthetic record is one part of perioperative anesthesia management that helps for medico-legal issues, to maintain a safe practice of anesthesia and for research purposes [14].

In developed countries the anesthesia record is performed electronically using the Anesthesia Information Management System and the completeness is continually assessed [15]. However, a manual paper-based recording is performed in most developing countries like Ethiopia. The current study tried to assess the completeness of perioperative anesthesia records in governmental hospitals of Amhara Regional State using parameters listed in a currently used anesthesia record sheet. In this study, we have used the recommendation of the American Association of Nurse Anesthetists that states the anesthesia records of any parameters should be completely recorded and any records with less than 95% were considered poor [10]. The completeness of the anesthesia record regarding patient identifiers was poor. The recording of patient weight was poor (43.1% of the records). In line with the current study, a study done in South Africa by M Raff and MFM James showed that the completeness of patient identifiers was low [13].

In this study, the completeness of all the documented data related to basic preoperative history was poor (less than 80% of the records). The completeness of the anesthesia record was poor in most of the parameters related to preoperative physical examination and the record completeness of basic preoperative investigations was also not adequate (less than 75% of the records). A study done in American Massachusetts General Hospitals showed 86% had at least one free text entry documenting the ECG rhythm diagnosis. Of the 2384 records in the study period containing any IV access documentation was only 17% of the records [2]. Another study in Canada showed the completeness of anesthesia charting remained low (<37%) [9]. The possible reasons for the incompleteness of anesthetic record sheets might be the overload of anesthetists and the anesthetists’ awareness towards the importance of completing the anesthetics record sheet.

The main limitation of this study is that the data was extracted from the anesthetic record sheets of the Amhara regional state hospitals of Ethiopia, which may vary from institution to institution.

## 5 Conclusion

The completeness of perioperative anesthesia record was poor in Amhara regional state hospitals of Ethiopia. Anesthesia professionals should document all the necessary perioperative parameters in the anesthesia record sheet.

## Ethical approval

Ethical clearance was obtained from the College of Health Sciences Research and Community Service Coordination Ethical Review Committee of Debre Tabor University with reference number Ref CHS/33102/2021.

## Authors’ contributions

Moges Gelaw Taye developed the proposal, collected the data, analyzed the data, and prepared the manuscript. Efrem Fenta, Tadese Tamire and Yewlsew Fentie involved in data collection, data analysis and manuscript preparation. All authors read and verify the manuscript for publication.

## Funding

10.13039/501100022150Debre Tabor University.

## Registration of research studies

1. Name of the registry: http://www.researchregistry.com.

2. Unique Identifying number or registration ID: researchregistry7644.

3. Hyperlink to your specific registration (must be publicly accessible and will be checked): https://www.researchregistry.com/browse-the-registry#home/?view_2_sort=field_21|asc.

## Guarantor

Mr. Yewlsew Fentie.

## Data sharing statement

Data will be shared from the corresponding author upon reasonable request.

## Consent for publication

Not applicable.

## Provenance and peer review

Not commissioned, externally peer-reviewed.

## Declaration of competing interest

The authors have declared that no competing interests.
